# The continuous and changing impact of affect on risky decision-making

**DOI:** 10.1038/s41598-022-14810-w

**Published:** 2022-06-23

**Authors:** Erkin Asutay, Daniel Västfjäll

**Affiliations:** 1 Department of Behavioral Sciences and Learning, Linköping University, 58183 Linköping, Sweden; 2 Decision Research, Eugene, OR USA

**Keywords:** Psychology, Human behaviour

## Abstract

Affective experience has an important role in decision-making with recent theories suggesting a modulatory role of affect in ongoing subjective value computations. However, it is unclear how varying expectations and uncertainty dynamically influence affective experience and how dynamic representation of affect modulates risky choices. Using hierarchical Bayesian modeling on data from a risky choice task (N = 101), we find that the temporal integration of recently encountered choice parameters (expected value, uncertainty, and prediction errors) shapes affective experience and impacts subsequent choice behavior. Specifically, self-reported arousal prior to choice was associated with increased loss aversion, risk aversion, and choice consistency. Taken together, these findings provide clear behavioral evidence for continuous affective modulation of subjective value computations during risky decision-making.

## Introduction

Affect and emotions have a central role in judgment and decision-making. Previous studies have demonstrated affective modulation of decision-making under risk and uncertainty^1,2^. The recent theories suggest that affective processes modulate information processing and decision-making in a context dependent manner, wherein affective feelings are used as information by the decision-maker, influencing the ongoing subjective value computations^1,3^. However, despite an abundant literature on affect and decision-making, there is still a lack of understanding of the mechanisms for the continuous modulation of decision-making by affective experience. The relationship between affect and decisions is complex because affective experience not only influences perception of decision options and subjective value computations but also is dynamic and continuously influenced by the encountered information in a decision context. Here, using a risky decision-making task and hierarchical Bayesian modeling, we investigated how self-reported affective experience fluctuates as a function of varying choice variables and how trial-to-trial variations in subjective affective experience influence risky decision-making in a dynamic context.

Most evidence on the impact of affect on risky choice comes from mood induction studies. Previous investigations have shown that anger and fear were associated with a subsequent increase and decrease in risk taking, respectively^4^. However, the opposite effects of anger and fear on risk taking were found depending on the decision context^5^ or when the task involved estimating a social instead of a monetary risk^6^. Yet, others found that anger leads to more optimistic risk estimations only in men but not in women^7^. On the other hand, investigations linking physiological indices of affect to risky decision-making have shown that anticipatory physiological arousal indexed by skin conductance responses is associated with less risky choices^8^. Additionally, increased autonomic arousal was associated with decreased risk taking especially when probability of winning was small^9^. This pattern of results leaves us with open questions about the role of affective experience on risk taking and suggest that the involvement of affect on risky choice is context dependent.

Two decision parameters, risk sensitivity and loss aversion, are often used to explain differences in risk preferences^10^. Risk sensitivity determines whether the decision indicates a risk-seeking or a risk-averse behavior, whereas loss aversion is the tendency to weigh potential losses more heavily than equivalent potential gains. Research has shown that higher arousal responses to losses relative to gains correlates with the degree of individual loss aversive behavior^11^. However, risk preferences are often unstable and context dependent^1^. Additionally, previous research has mainly focused on average risk preferences at the individual or group level, and not so much on the intraindividual variations in risky decision-making as a result of ongoing affective processing. Thus, there are still unanswered question about how affect continuously modulates risky choice in a dynamic context. Given the continuous nature of affect, there is a need to adopt an experimental framework attempting to capture the key dynamic parameters of how moment-to-moment affective fluctuations modulate subjective value computations in risky choice.

Affect is a stream of fluctuations in an organism’s neurophysiological state representing its ongoing relationship with the environment^12,13^. Hence, it is a continuous and temporally dependent mental process. Previous research has shown that momentary affective experience is shaped by a temporal integration of the currently active information and previously experienced affect^14,15^. Furthermore, it has been shown that moment-to-moment happiness ratings during risky decision-making depends on the temporal integration of the affective impact of previous expectations and prediction errors^16^. However, the temporally dependent nature of affect is often not considered when studying its role in decision-making. In most studies, researchers use paradigms, in which individuals make a series of randomized, independent choices between different options. Then, the choice behavior is often modeled based on the given information and random noise^17^, while affect being operationalized either as induced mood states that are assumed to be static and long-lasting or as stable individual difference measures. This is at odds with the temporally dependent and dynamic structure of mental processes like affect^18^. Hence, there is a need to model affect dynamics reflecting the temporal integration of previously encountered events to understand how moment-to-moment variations in affective experience influence risky decision-making. The purpose of the current study is to investigate (1) affective fluctuations as a function of varying expectations, uncertainty, and prediction errors and (2) the variation in subsequent risky decision-making as a result of these ongoing affective fluctuations. We used a novel risky decision-making task, in which individuals make a series of risky monetary decisions and report their momentary affective experience. We adopted a computational modeling approach to capture the key dynamic variables determining affective experience based on previously encountered choice variables (i.e., expected value, uncertainty, and prediction error). Moreover, we modeled the influence of trial-to-trial variations in affective experience on decision parameters of loss aversion, risk sensitivity, and choice consistency.

## Method

### Participants

We recruited 108 individuals through a university participant pool. The study was run online using Inqusit (Inqusit version 5) and participants were compensated after the study. Each participant received a 75 SEK (approx. $7.5 at the time of the study) participation fee. In addition, one of the decisions for each participant was selected randomly and played out for real at the end of the study. The outcome of this gamble defined the final compensation (Mean = 80 SEK, SD = 14 SEK). Data from seven individuals were excluded from all analyses. One participant provided the same response in more than 80% of the trials. Six other participants were removed because they provided affect ratings without moving the scale sliders in more than 80% of the trials. Hence, the final sample consisted of 101 individuals (34 females, Mean age = 24, SD = 5.2). The study was conducted in accordance with the ethical standards in the Declaration of Helsinki and approved by the Swedish ethical review authority. Formal power analysis was not conducted as we used Bayesian analyses. The data collection was open for 2 weeks after which we stopped the collection and analyzed the data. Data and modeling codes are publicly available (https://osf.io/ryfu9/). The modeling was done using Stan^19^ and fitted in R using *rstan* package^20^. The study design and analyses were not pre-registered.

### Risky decision-making task

Participants viewed 50 monetary gambles each consisting of four possible outcomes (gains and/or losses) with associated probabilities. The possible outcomes of a given gamble are selected randomly from a normal distribution (Mean = 0SEK, SD = 18SEK) and rounded to the nearest 5 SEK (e.g., 13.4 ~ 15; and − 7.9 ~  − 10). In addition, we apply the following limitations: (1) the maximum possible loss or gain will not exceed 75 SEK, (2) all four possible outcomes of a given gamble will be different, (3) ‘0 SEK’ will not be a possible outcome, and (4) the expected value of any gamble will not exceed ± 25 SEK. When generated in this way, expected values come from a normal distribution with 0 SEK mean and 10 SEK standard deviation (for details, see Supplementary Information). The monetary gambles were generated separately for each participant. With this novel choice set, we aimed to test a method to study risky decision-making, wherein the distribution of possible outcomes is defined rather than a predetermined choice set every participant goes through.

The following independent choice variables were extracted from each gamble: expected value of the gamble (EV), uncertainty around the EV (U), and prediction error (PE). EV is determined as

1 $$ EV = \mathop \sum \limits_{i}^{4} x_{i} *p_{i} $$

where *x*_*i*_ is a monetary outcome and *p*_*i*_ is its probability. Uncertainty (U) is given by the variance of the EV.

2 $$ U = \mathop \sum \limits_{i}^{4} \left( {x_{i} - EV} \right)^{2} *p_{i} $$

*U* was square-root transformed to reduce the skewness inherent in the variable, and centered around zero. PE is given as the difference between the actual outcome and EV.

3 $$ PE = \left\{ {\begin{array}{*{20}l} {Outcome - EV,} & {\quad if\,gamble\, is \,accepted} \\ {0, } & {\quad if\,gamble\, is \,rejected} \\ \end{array} } \right. $$

### Experimental procedure

**Figure 1:**
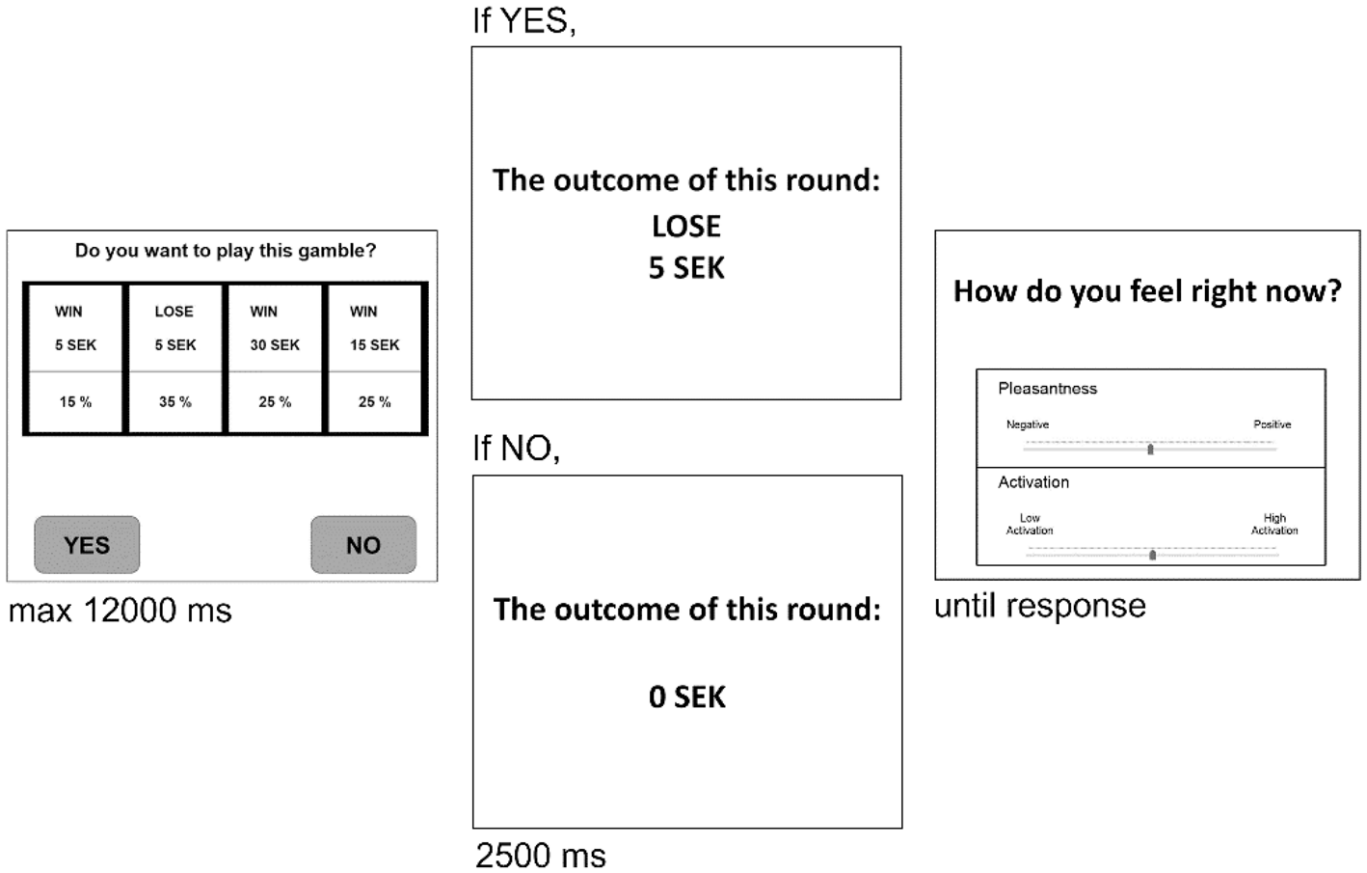
Trial structure. Participants viewed a gamble with four possible outcomes and decided whether to accept it or not, after which they received feedback. Following the feedback, they were asked to report how they felt using pleasantness and activation scales.

Each participant, after reading the instructions and giving informed consent online, went through the gambles. In each trial, participants viewed the given gamble and decided whether to accept or reject it (Fig. 1), after which participants received feedback regarding the outcome of the trial. Participants viewed the outcome of the gamble if they chose to play it; otherwise, they viewed “*0 SEK*” as the outcome. Following the feedback, participants reported how they currently feel on visual analog scales of valence (unpleasant to pleasant) and arousal (sleepiness to high activation). Participants were explicitly instructed to assess how they currently feel at the time of reporting. Valence and arousal ratings were individually standardized (i.e., z-score) to represent each individual’s data in terms of their own means and variations.

### Data analysis

#### Affective experience

As a manipulation check, we analyzed whether valence and arousal ratings were impacted by positive and negative trial outcomes. We used generalized linear mixed models (using *fitglme* function in Matlab version R2019b) to predict valence and arousal ratings based on experienced gains and losses. Both valence and arousal models included fixed effects of the outcome experienced in the current trial, together with random intercepts and slopes at the individual level which means that both the intercepts and the estimates of the predictors were allowed to vary among individuals.

#### Choice behavior

We carried out a series of generalized logistic mixed models (using *fitglme* function in Matlab version R2019b) to ensure that participants understood the novel risky choice task and that choice behavior reflected the possible outcomes of a gamble and not just the extremes. The main model included a fixed effect of the gamble EV. We also tested alternative models including fixed effects of (1) the best (i.e., the biggest gain) and the worst (i.e., the biggest loss) possible outcomes, (2) the best and the worst possible outcomes weighted with their respective probabilities, and (3) the outcome with the highest probability of occurring. All models included random intercepts and slopes at the individual level. The model fits were assessed using Akaike information criterion (AIC).

### Computational modeling

We used hierarchical Bayesian analysis (HBA) to study affective fluctuations as a function of varying decision variables and the potential influence of this changing affective experience on risky decision-making. HBA estimates posterior distributions that reflect uncertainty for parameters at group and individual levels and optimizes the tradeoff between random and fixed-effects models of individual differences^21–23^. Individual participants are constrained by group distributions, but they can also vary from the group distributions to the extent their data are diagnostic.

#### Affective experience

We have used a previously established model that generates a momentary subjective affective state based on the integration of previously encountered information^16,24^. Trial-by-trial affective experience is generated based on an exponential decay of the influences of previous events. We tested alternative models with outcomes instead of expectations and prediction errors, with the uncertainty term, and with different parametrizations of the prediction error term (see Supplementary Information, Table S1). The model with the best fit for both valence and arousal ratings included terms for EV, U, PE, and the magnitude of PE according to widely applicable information criterion (WAIC), a Bayesian approach for estimating out-of-sample predictive accuracy with lower values indicating a better fit^23,25^.

4 $$ \begin{aligned} AE_{t,i} & = w_{0,i} + w_{EV,i} \mathop \sum \limits_{j = 1}^{t} \gamma_{i}^{t - j} *EV_{j,i} + w_{U,i} \mathop \sum \limits_{j = 1}^{t} \gamma_{i}^{t - j} *U_{j,i} \\ & \quad + w_{PE,i} \mathop \sum \limits_{j = 1}^{t} \gamma_{i}^{t - j} *PE_{j,i} + w_{{\left| {PE} \right|,i}} \mathop \sum \limits_{j = 1}^{t} \gamma_{i}^{t - j} *\left| {PE_{j,i} } \right| \\ \end{aligned} $$

*AE*_*t,i*_ is affective experience (valence and arousal) for individual *i* at time point *t*. *EV*_*j,i*_, *U*_*j.i*_, and *PE*_*j.i*_ are decision variables in trial *j*. The free parameters of the model in are *w* and *γ* terms determining the affective integration. The *w*_*0,i*_ is the constant term representing a baseline affective experience around which an individual fluctuates during the task. The parameters *w*_*EV,i*_, *w*_*U,i*_, *w*_*PE,i*_, *w*_*|PE|,i*_ are the weights on the decision variables *EV*, *U*, *PE*, and the magnitude of PE (i.e., absolute value of PE), representing the degree to which these variables impact an individual’s affective experience. We included the PE magnitude as a variable in the model to test whether especially the arousal feature of affect is sensitive to the size of the PE regardless of its direction. Finally, *γ*_*i*_ is an individual forgetting factor adjusting the influence of recent events in comparison to earlier events with *0* ≤ *γ* ≤ *1*. This parameter defines the relative impact of earlier vs. later stimuli on momentary affect. As *γ* approaches to 1, each trial is weighted evenly, while a *γ* of 0 means that only the current trial determines the current affective experience. Since the affective ratings are standardized, the model cannot distinguish mean level differences between participants, which effectively impacts the constant parameter (*w*_*0*_). However, standardization does not influence trial-by-trial changes, therefore, the rest of the parameters defining the affective integration can be estimated reliably.

**Figure 2:**
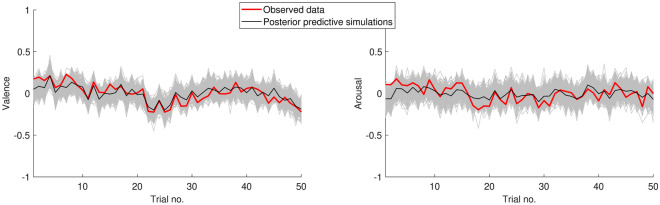
Posterior predictive checks for the affect model. Average valence and arousal over the experiment are shown (solid red) together with 500 posterior predictive simulations from the affective experience model (grey area) and the median predictions (solid black).

We used HBA to estimate group and individual level parameters separately for valence and arousal ratings. The parameters for each individual were modeled to be distributed according to a normal (the weight parameters), beta (forgetting factors), or lognormal (individual variance) centered around a group mean with a group variance with weekly or non-informative priors (for details on priors and fitting procedure, see the Supplementary Information). The model was coded in Stan^19^ and fitted in R using the *rstan* package^20^. Markov chain Monte Carlo (MCMC) sampling methods were used to estimate the posterior distributions of the parameters. We fit the model separately to valence and arousal ratings using 8 sampling chains for 3000 iterations each, 1000 of which were discarded as warm-up samples. This resulted in 16,000 samples for each parameter. Trace plots of the group level parameters were visually inspected to ensure convergence. We also ensured that all $\widehat{R}$ values were under 1.01 which indicates that chains have adequate mixing^26^. Additionally, posterior predictive checks showed that the models captured the overall valence and arousal fluctuations (Fig. 2).

#### Choice behavior

##### Base model

We used a choice model based on prospect theory to parametrize participants choice behavior based on subjective utility computations.

5 $$ \begin{array}{*{20}l} {u_{i} = x_{i}^{\rho } } \hfill & {\quad if\,\, x_{i} > 0} \hfill \\ {u_{i} = - \lambda \left( { - x_{i} } \right)^{\rho } } \hfill & {\quad if\,\, x_{i} < 0} \hfill \\ \end{array} $$

6 $$ V_{gamble} = \mathop \sum \limits_{i = 1}^{4} u_{i} .p_{i} $$

Equation (5) shows the utility calculation of a possible monetary outcome, *x*_*i*_, with two free parameters, *λ* and *ρ*. *λ* determines the asymmetric weighting of gains and losses and is also known as loss-aversion, a central tenet of prospect theory with *λ* > *1* indicating loss aversive behavior^10^. *ρ* reflects the curvature of the value function in prospect theory and reflects diminishing or increasing marginal utility. *V*_*gamble*_ in Eq. (6) represents the subjective value of a gamble, which is the probability (*p*_*i*_) weighted sum of the utilities of the four possible outcomes. The probability of accepting the risky gamble then depends on *V*_*gamble*_.

7 $$ p\left( {risk} \right) = \frac{1}{{1 + e^{{ - c . V_{gamble} }} }} $$

Here, *c* (*0* ≤ *c* ≤ *20*) is choice consistency (i.e., inverse temperature) parameter that captures randomness in participants choices, in which *c* = *0* indicates fully random decisions.

Similar to the affect model, we used HBA to estimate group and individual level parameters. The parameters (*λ*, *ρ*, and *c*) for each individual were modeled to be distributed around a group mean with a group variance. We used a non-centered parametrization to speed up estimation^20,27^ and to avoid invoking hard bounds on parameters (for details on priors and fitting procedure, see the Supplementary Information). In addition, we have carried out a parameter recovery analysis to ensure that the novel risky choice task and the modeling procedure produce reliable parameter estimates under ideal conditions (for details, see Supplementary Information). We generated 100 datasets (N = 101 per group) with a random selection of group level means and variances for the model parameters (*λ*, *ρ*, and *c*). The results indicated that both group level means (100 simulations) and individual parameters (10,100 simulated individuals) for *λ* and *ρ* could be recovered independently. *c* was overestimated in 30% of the simulations when the data generating group level c was larger than 2, and the group level *ρ* was larger than 1. However, group level *c* below 2 were reliably recovered (see Figures S3, S4, & S5 in the Supplementary Information). Taken together, parameter recovery analysis suggests that the current task design and analysis may be limited when the data generating process is defined by an increasing marginal utility together with a high choice consistency among participants.

##### Trial-by-trial variations

To test our hypothesis that changing affective experience depending on varying decision variables dynamically influences risky decision-making, we implemented regression coefficients by reparametrizing the model parameters (*λ*, *ρ*, and *c*) so that they were influenced by trial-by-trial variations of valence and arousal. We estimated posterior distributions for the degree to which *λ*, *ρ*, and *c* were altered by the variations in momentary affective experience. Below is a formulation of trial-by-trial affective fluctuations for the loss aversion parameter.

8 $${\lambda }_{i,t}={\lambda }_{i}+{\beta }_{V,i}.{Valence}_{i}\left(t\right)+{\beta }_{A,i}.{Arousal}_{i}\left(t\right)$$

The regression coefficients, *β*_*V,i*_ and *β*_*A,i*_, reflect the degree to which loss-aversion changes with experienced valence and arousal before the current decision (i.e., reported at the end of the previous trial), and they are modeled to be distributed around a group mean with a group variance (for details on fitting and priors, see Supplementary Information). *λ*_*i*_ in Eq. (8) is the average loss aversion for participant *i* and is drawn from the group-level distribution as in the base model above. *λ*_*i,t*_ is the trial-by-trial loss aversion fluctuating around the individual average, and these fluctuations are determined by the individual’s affective experience. We implemented equivalent regressions for the other parameters (*ρ and c*).

##### Model fitting and comparison

We first fitted the base model without including affective experience data. Next, we tested whether trial-by-trial variations in affective experience influence decision parameters. We formulated a full model, in which self-reported valence and arousal before a trial influence all the decision parameters. We then systematically removed regressors that did not reliably modulate the decision parameters to test whether the model fit was improved. Model comparisons were done according to WAIC to approximate how well each model would perform on new participants. Thus, WAIC was computed across participants, where the log-likelihood for each participant’s choice data were summed across trials (for the model fit of all the tested models, see Supplementary Information). We found that the full model with regressors performed better than the base model without the influence of affective experience (WAIC_Base_ = 2714, WAIC_Full_ = 2698). According to the full model, variations in valence did not reliably influence any decision parameters, while arousal was positively associated with *λ* and *c*, and negatively associated with *ρ* (Figure S2 & S3 in Supplementary Information). Next, we removed the valence modulators from the full model to see whether the model fit would improve, which resulted in a slightly lower information criterion (WAIC_Adjusted_ = 2694). Since both arousal and valence are influenced by gamble outcomes, we have formulated two alternative models, in which we studied the arousal effect while controlling for the trial outcome and PE from the previous trial. In these models, the trial outcome and PE are used as additional regressors modulating trial-by-trial decision parameters. These models indicated that the arousal effect persisted, while the influence of gamble outcome and PE were not significantly different from zero (see Table S3 in Supplementary Information).

Each choice model was coded in Stan^19^, fitted in R using the *rstan* package^20^ with 8 sampling chains for 3000 iterations each, 1000 of which were discarded as warm-up samples (16,000 samples in total). Trace plots of the group level parameters were inspected for convergence and $\widehat{R}$ values were ensured to be under 1.01^26^.

## Results

### Behavioral results

#### Affective experience

First, we studied the immediate impact of losses and gains on valence and arousal ratings as a manipulation check. We found that gains (B = 0.46, 95% CI [0.44, 0.48], *p* < 0.001) and losses (B =  − 0.47, 95% CI [− 0.44, − 0.5], *p* < 0.001) were positively and negatively associated with experienced valence. The arousal analysis showed that both gains (B = 0.32, 95% CI [0.29, 0.35], *p* < 0.001) and losses (B = 0.19, 95% CI [0.15, 0.23], *p* < 0.001) were positively associated with experienced arousal.

#### Choice behavior

We carried out logistic mixed models to ensure that participants’ choice behavior reflected the outcomes and their probabilities, and not just the best and the worst possible outcomes. We tested four different logistic mixed models. The model containing the gamble EV as a fixed effect clearly outperformed all the other models (AIC_EVmodel_ = 2972; AIC_alternative1_ = 4810; AIC_alternative2_ = 4746; AIC_alternative3_ = 4974), indicating that choice behavior reflected a combination of outcomes and probabilities rather than just the best and worst possible outcomes. The parameter estimates showed that gamble EV was positively associated with risk taking (log Odds-Ratio = 0.33, 95% CI [0.31, 0.35], *p* < 0.001).

### Affective experience model

We investigated the influence of varying expectations, uncertainty, and prediction errors on affective experience using a computational model aimed at capturing momentary changes in subjective affective states. The model was fit to valence and arousal data with hierarchical Bayesian estimation, which optimizes the tradeoff between random and fixed-effects models of individual differences. Table 1 summarizes the model parameters with the posterior distribution of the group level means and reports 95% highest density intervals (HDIs) indicating the ranges, in which the most probable 95% of values fall.

**Table 1:** The posterior distributions on parameter estimates showing the group level means of parameters.

Parameters	Valence model	Arousal model
*w*_*0*_	0.03 [− 0.01, 0.07]	− 0.23 [− 0.34, − 0.13]
*w*_*EV*_	0.26 [0.23, 0.3]	0.21 [0.17, 0.24]
*w*_*U*_	− 0.04 [− 0.09, − 0.01]	0.06 [0.01, 0.1]
*w*_*PE*_	0.49 [0.46, 0.53]	0.15 [0.12, 0.19]
*w*_*|PE|*_	− 0.04 [− 0.07, − 0.01]	0.16 [0.12, 0.21]
*γ*	0.25 [0.18, 0.33]	0.49 [0.38, 0.6]

The ranges in parentheses represent 95% Highest density intervals (HDIs).

The 95% HDI of ***w***_***EV***_ indicates that EV of the gambles had a positive impact on both valence (95% HDI = [0.23, 0.3]) and arousal (95% HDI = [0.17, 0.24]). Furthermore, ***w***_***U***_ was negative for valence (95% HDI = [− 0.09, − 0.01]) and positive for arousal (95% HDI = [0.01, 0.06]), which indicates that uncertainty leads to negative valence and increased arousal. We also found that prediction errors had positive impact on valence (95% HDI = [0.46, 0.53]) and arousal (95% HDI = [0.12, 0.19]). In addition, the PE magnitude independent of its direction was associated with negative valence (95% HDI = [− 0.07, − 0.01]) and increased arousal (95% HDI = [0.12, 0.21]). These estimated weight parameters associated with PE indicate that valence fluctuates with the direction of PE; that is, positive surprises cause positive affect and negative surprises cause negative affect. Whereas, arousal feature of affect is impacted by both the magnitude and the direction of PE, which means a large surprise (whether it is positive or negative) has a potential to increase arousal. Furthermore, the fact that ***w***_***|PE|***_ was negative for valence and positive for arousal indicates that positive PEs had a smaller effect on valence and a larger effect on arousal compared to negative PEs.

Moreover, ***w***_***PE***_ was larger than ***w***_***EV***_ for the valence model indicating a stronger influence of prediction errors in comparison to the expected value, an effect reported by previous studies^16,24,28^. Finally, the posterior distribution of the forgetting factors showed lower values for valence (95% HDI = [0.18, 0.33]) in comparison to arousal (95% HDI = [0.38, 0.6]), suggesting that the impact of earlier trials was stronger for arousal than for valence. Taken together, these results show that the affective impact of expected value, the uncertainty around the expected value, and prediction errors are temporally integrated in momentary affective experience with differential impacts on valence and arousal.

### Choice model

We fit the choice model with and without the trial-by-trial affective modulations of decision variables (Figure S6 & S7 in Supplementary Information). The model comparison showed that the model with affective regressors performed slightly better than the base model without the affective influence. Results showed that variations in valence did not reliably influence any decision parameter, while the influence of arousal was reliably different from zero evidenced by the 95% HDIs (Figure S7 in Supplementary Information). Finally, we removed the valence modulators from the full model, which resulted in a slightly better model fit.

**Figure 3:**
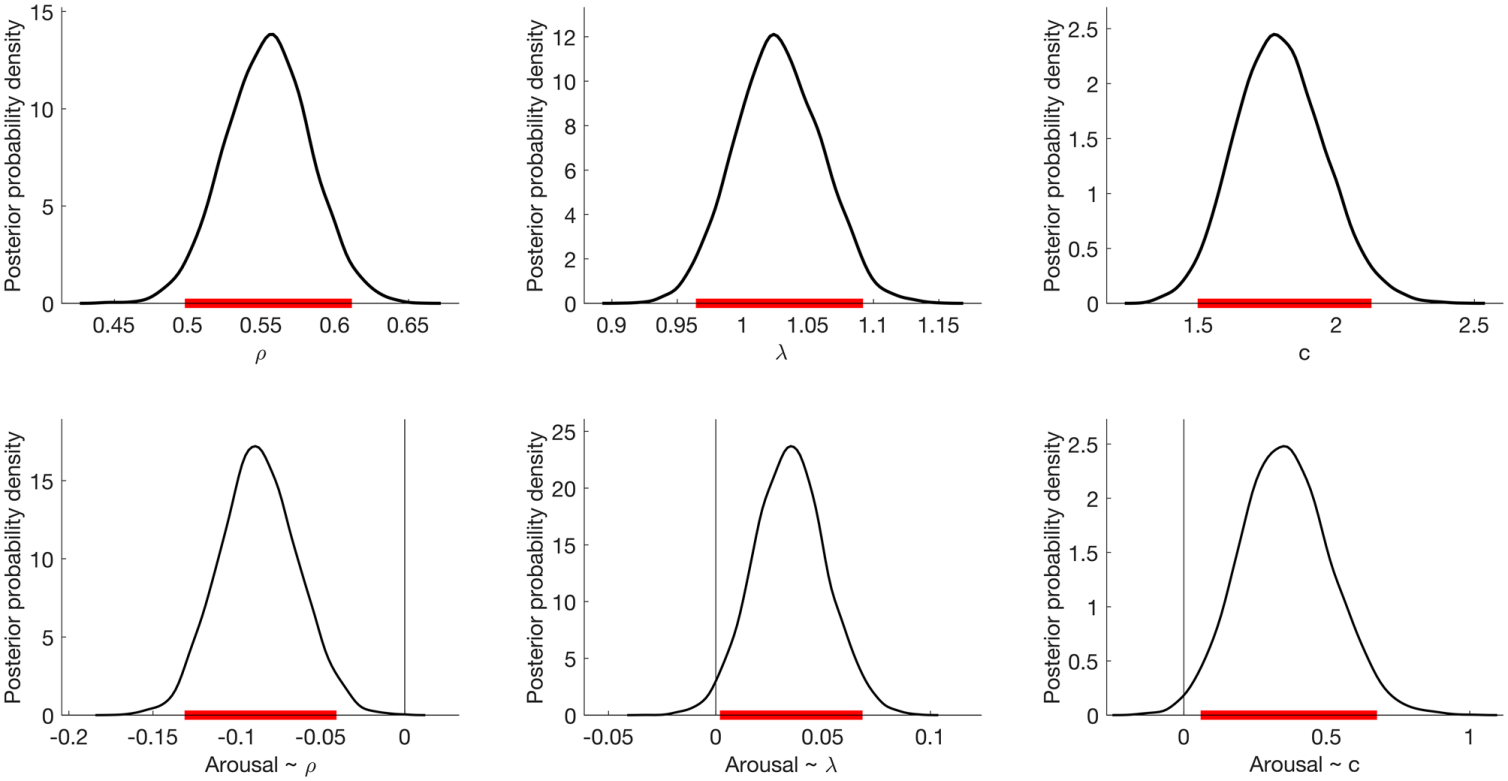
The posterior distributions on model parameter estimates showing the group level means and trial-to-trial influences of arousal on decision parameters. The upper panel shows the posterior distributions and the 95% HDIs for group level parameters (marked ranges under the distributions). The lower panel shows the posterior distribution of the regression coefficients. The peak value of each distribution represents the best estimate, while the width represents the uncertainty of the estimate.

Here, we summarize the final adjusted model and report 95% HDIs of the posterior distribution of the group level means (Fig. 3). We found that participants choices demonstrated diminishing marginal utility (95% HDI on *ρ* = [0.5, 0.61]). In addition, there was evidence against loss aversion as the 95% HDI on *λ* included 1 (95% HDI on *λ* = [0.96, 1.09]). Finally, participants were consistent in their choices (95% HDI on *c* = [1.49, 2.13]). This model also included the impact of trial-by-trial arousal variations on the decision parameters. Interestingly, arousal was negatively associated with *ρ* (95% HDI on ${\beta }_{A}\propto \rho$ = [− 0.13, − 0.04]) and positively associated with both *λ* (95% HDI on ${\beta }_{A}\propto \lambda$ = [0.01, 0.07]) and *c* (95% HDI on ${\beta }_{A}\propto c$ = [0.06, 0.68]). Taken together these estimates indicate that variations in experienced arousal influence risky decision-making by modulating subjective value computations and choice consistency. The findings suggest that increased arousal leads to a slightly increased loss averse and risk averse behavior as well as increased choice consistency in subsequent risky choices. Importantly, these effects of arousal were confirmed when controlling for the outcome or PE from the previous gamble (see Table S3 in Supplementary Information).

## Discussion

The current study set out to investigate how varying expectations, uncertainty, and prediction errors influence continuous affective experience, and how this dynamic representation of affect modulates ongoing subjective value computations during risky decision-making. Momentary affect was measured using self-reports of experienced valence and arousal. The results from the computational model of affect revealed that expected value, uncertainty, and prediction errors were temporally integrated into overall affective experience with differential impact on experienced pleasantness and arousal. Furthermore, we estimated the decision parameters allowing for them to change with affective experience prior to choice to assess whether subjective value computations are modulated by momentary affect. We found that experienced arousal prior to choice was associated with increased loss aversion and risk aversion, as well as higher choice consistency, whereas trial-by-trial valence did not reliably influence decision parameters. These findings present clear behavioral evidence for continuous affective modulation of subjective value computations during risky decision-making.

The posterior distribution of parameter estimates of the computational affect model indicated that both expected value and prediction errors were positively associated with valence and arousal. Previous investigations using the same mathematical model found that moment-to-moment happiness ratings were partly shaped by expected value and prediction error, with significantly stronger impact of the latter^16,24,28^. The current evidence on the impact of expected value and prediction error on valence ratings are in line with these previous findings. Moreover, our results indicate that it is possible to use a similar model to study moment-to-moment arousal as a function of previously encountered events. Critically, we also have shown that uncertainty of the expected value can also be integrated in the same model. Importantly, uncertainty was associated with negative affect and increased arousal, which is in line with previous findings showing that uncertainty can cause unpleasant affect^15,29^ and anxiety^30^, and that uncertainty signals from the environment drive the physiological arousal systems^31,32^. The results also indicated that prediction error magnitude independent of its direction was associated with increased arousal and slightly unpleasant affect. We additionally found that forgetting factor was larger in arousal relative to valence, which indicates that the affective impact of previous events is stronger on arousal in comparison to valence. This also means that valence fluctuates more closely with the current stimuli compared to arousal, which is in agreement with previous findings from studies with affective images^15^. Taken together, the temporally sensitive modeling approach we adopted proves a useful strategy for revealing the differential dynamics of valence and arousal features of affective experience depending on varying expectations, uncertainty, and prediction errors during risky decision-making.

The computational modeling of choice behavior revealed that allowing the decision parameters to change with affective experience improved the model fit. This suggests that subjective value calculations and choice consistency are modulated by momentary variations in affective experience. We found that arousal, but not valence, reported in the previous trial was associated with increased loss aversion, risk aversion, and choice consistency for the subsequent decision. Previous research has reported a positive association between arousal and loss aversion at an individual level; that is, loss aversive behavior is associated with higher arousal responses and increased amygdala activations to losses relative to gains^11,33^. The current findings on the association between arousal and loss aversion is critically different; that is, intraindividual variations in loss aversive behavior is modulated by momentary arousal. Recent findings indicate that trial-to-trial outcomes influence subsequent risky decision-making despite the traditional assumption that the trials are independent from one another^34,35^. Positive previous outcomes in risky monetary decisions can increase loss aversion and choice consistency for subsequent choices^34^. These contextual influences suggest that risky decision-making is fundamentally dynamic and temporally dependent on the impact of previous events. Affect, reflecting the individual’s ongoing relationship with the environment, represents the cumulative impact of previous events together with future predicted states^12,13,18^. Hence, it is a critical mechanism through which recent events may influence subsequent behavior. The effects we identified provide evidence for the underlying affective correlates of the impact of recent events on subsequent risky choices. Our findings clearly show that affect acts as a summary of recent prediction errors, decision uncertainty, and expectations, and that momentary changes in arousal temporarily modulates subjective value computations and choice consistency.

We find that subjective arousal encodes varying expectations, uncertainty, and predictions errors (both magnitude and direction) and influences subsequent risky choice by modulating ongoing subjective value computations. Previous investigations suggest that arousal may be driven by the changes in uncertainty due to changes in environmental signals^32^. It was shown that the extent of the correlation between uncertainty and arousal predicts individual performance in probabilistic learning^31^. Moreover, decision uncertainty in a perceptual decision task may lead to rapid changes in pupil-linked arousal, which in turn shapes the choice behavior in subsequent trials^36^. Additionally, in risky choice, arousal is associated with anticipation of risks^8,9^. Hence, these earlier findings together with the current results suggest that arousal represents the changes in uncertainty signals together with prediction errors and modulates ongoing behavior (see also,^37^).

On the other hand, trial-by-trial valence ratings did not modulate decision parameters. Previous research has reported significant effects of emotional valence on risk taking (e.g.,^4,7,38,39^). Most of this evidence comes from mood induction procedures, in which affect is manipulated through stimuli and procedures strictly incidental to the task aiming to induce specific emotional states (e.g., anger, fear, depression, excitement, anxiety). We argue that these induced states are much more complex and longstanding emotional states compared to momentary affect, which is low-dimensional. Thus, we interpret the current effects we report as momentary affective fluctuations due to varying expectations, uncertainty, and prediction error signals temporarily modulating subjective value calculations. We report that these signals differentially impact experienced valence and arousal. Given that arousal, but not valence, had a modulatory influence on decision parameters, the affective influence of varying uncertainty and prediction error magnitude may be responsible for the current findings.

The current study presents a novel risky choice task, in which the choice sets are selected from a distribution of possible outcomes and probabilities instead of a predetermined choice set for all participants. One potential limitation for the current approach is that the participants do not go through the same choice set, which may be problematic for parameter estimation if the range of outcomes widely vary between participants. However, the choice sets in our study covered the same outcome ranges and EV distributions and did not include extreme outcomes (see Supplementary Information). Moreover, the affect model parametrizes experienced affect as a temporal summary of previously encountered events. Thus, for a given trial, the choice options participants previously experienced will not be identical even for a fixed choice set, since there is often a need to randomize the trials to circumvent order effects. Hence, the critical contribution of our manuscript (i.e., the impact of previously experienced arousal on the current decision) should not be affected by whether the choice sets are identical or not. Finally, the studies validating the affect model had employed a risky choice task with a fixed choice set and reported similar results^16^. In our study, we extended this pattern to momentary pleasantness and arousal ratings and quantified the affective impact of decision uncertainty.

In addition, the size of the choice set in our study (i.e., 50 trials per person) is lower than most studies use to estimate the decision parameters. Even though the current use of hierarchical estimation techniques alleviates this potential issue to some extent, the limited set of choice options may have still influenced parameter estimation. Future studies using similar study designs may benefit from increasing the size of the choice set, which may further ensure that outcome ranges are similar between participants. Furthermore, the current approach does not guarantee that the choice set will include gain-only, loss-only, and mixed gambles, which may lead to loss aversion and risk sensitivity to be confounded. However, the parameter recovery analysis shows that both loss aversion and risk sensitivity can be reliably recovered over a reasonable range without being correlated with each other. This analysis validates our assumptions and suggests that the current risky choice task is suitable for the aims of the current study. Finally, we argue that generating choice sets from a distribution of outcomes may be used in future investigations studying how risky decision-making parameters change and adapt as the distribution of outcomes are varied.

We report reliable effects of previous affective experience on various decision parameters. It is, however, critical to note that momentary affective changes may also modulate risky decision-making through other paths that are not studied nor manipulated here. For instance, it was shown that anticipated affective states and emotions influence decision making^40,41^. In addition, counterfactual emotions (e.g., regret) stemming from a comparison between an actual outcome and what would have occurred under a different choice may be another path of affective modulation of choice behavior (e.g.^42^). The current risky choice task, following similar studies^16,24,28,34^, represents a context in which individuals experience only the direct outcome of their choices (not the counterfactual information). Future studies providing full feedback, in which participants see the outcomes of the rejected gambles, can be beneficial in studying the continuous affective impact of counterfactual information on risky decision-making. The anticipatory affect and counterfactual thinking may produce prediction error signals that may influence risky decision making through their specific affective impact.

Another potential limitation of the current study is the decision parameters not modeled in our study (e.g., probability weighting) potentially being influenced by variations in affective experience^43,44^. Even though it is reasonable to assume a linear weighing, not accounting for a possible non-linear probability weighting function may have impacted other parameters. The further development of temporally and contextually sensitive models including neural and physiological correlates of affect will be critical in understanding the continuous modulatory influence of affective processing on decision-making under risk.

The brain encodes the individual’s beliefs about the hidden states of the world^45^. These beliefs are probability distributions representing expectations and uncertainty. Hence, decision-making is about sampling the most appropriate action based on expected rewards and losses as well as the uncertainty around these expectations^46^. The current study provides clear behavioral evidence that ongoing affective experience encodes varying expectations and uncertainty that underlie our decisions, and it keeps track of the recent prediction error history. We have also shown that the arousal feature of this dynamic representation of affective experience, then, continuously modulates risky decision-making. Taken together, these findings point towards the benefit of adopting an experimental framework that attempts to understand the dynamic aspects of the involvement of affect in decision-making to uncover the mechanisms through which affect modulates our choices.

## Supplementary Information

Supplementary Information.

## Data Availability

All data and modeling codes are available at https://osf.io/ryfu9/.
