# Supplementary material for: The continuous and changing impact of affect on risky decision-making
Source: Sci Rep. 2022 Jun 23;12:10613. doi: 10.1038/s41598-022-14810-w (PMC9226037; doi:10.1038/s41598-022-14810-w)
Supplement: Supplementary file 1 — Supplementary Information. [file 41598_2022_14810_MOESM1_ESM.pdf]

Supplementary Information

The continuous and changing impact of affect on risky decision-making

Erkin Asutay[1, *] & Daniel Västfjäll[1, 2]

[1] Department of Behavioral Sciences and Learning, Linköping University, Sweden

[2] Decision Research, OR

Monetary gambles

The monetary gambles were generated individually. The possible outcomes of a given gamble were selected randomly from a normal distribution (mean = 0SEK, sd = 18SEK; 1SEK ~ $0.1) and rounded to the nearest 5 SEK (e.g., 13.4 ~ 15; and -7.9 ~ -10). Probabilities were selected from a truncated normal distribution (mean=0.25, sd=0.1) and rounded to the nearest 0.05, 0.15, 0.25, 0.35, or 0.45. Three probabilities were selected in this way and the fourth was calculated to ensure that the sum of the probabilities is 1. We then matched the monetary outcomes and probabilities randomly. Additionally, the following limitations were applied: (1) the maximum possible loss or gain will not exceed 75 SEK, (2) all four possible outcomes of a given gamble will be different, (3) '0 SEK' will not be a possible outcome for a given gamble, and (4) the expected value of any gamble will not exceed ±25 SEK. When generated in this way, expected values come from a normal distribution with 0 SEK mean and 10 SEK standard deviation (Figure S1). We also controlled that range of actual possible outcomes were similar across participants (Figure S2).

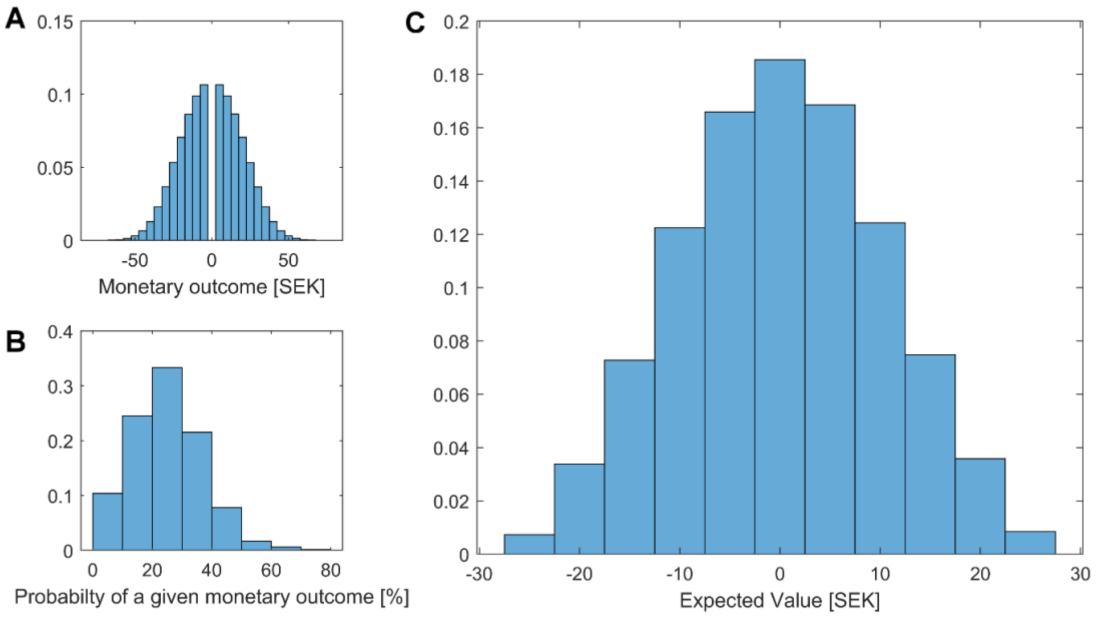

Figure S1. The distribution of monetary outcomes (A) and their probabilities (B). The monetary outcomes and probabilities are matched randomly. Each gamble has four possible outcomes. Panel C shows the distribution of expected value of gambles.

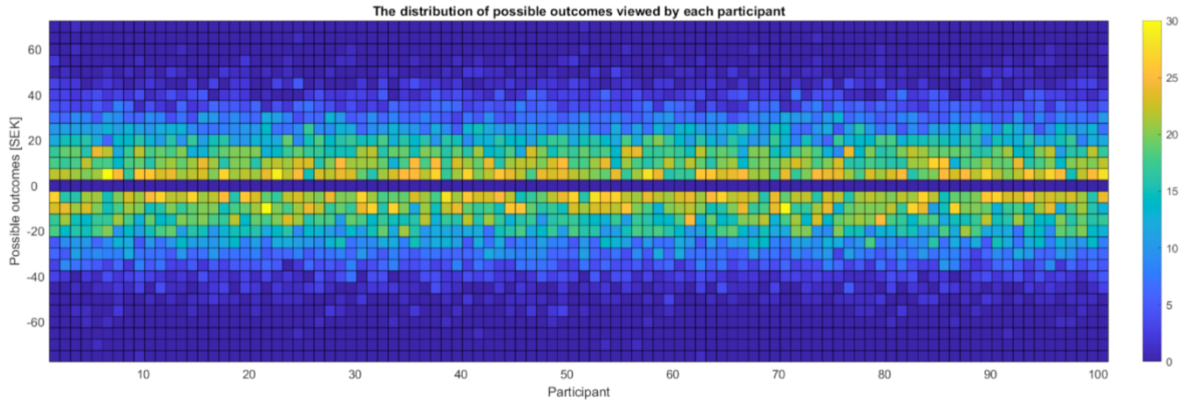

Figure S2. The distribution of monetary outcomes seen by each participant.

Affective experience model

The momentary valence and arousal responses were modeled using the exponential decay of the affective influence of previously encountered events. The models included terms for expected value (EV), uncertainty (U), and prediction error (PE; see main text for computation of the terms). We tested alternative models including models with outcomes instead of EV and PE, without uncertainty, or with different parametrizations of PE. Model 1 contained separate terms for gamble EV, and PE.

$$AE_{t,i} = w_{0,i} + w_{EV,i} \sum_{j=1}^{t} {\gamma_i}^{t-j} * EV_{j,i} + w_{PE,i} \sum_{j=1}^{t} {\gamma_i}^{t-j} * PE_{j,i} \quad \text{(S.1)}$$

$AE_{t,i}$ is affective experience (valence and arousal) for individual i at time point t. $w_{0,i}$ is the constant term. $w_{EV,i}$, and $w_{PE,i}$ capture the impact of expectations and prediction errors. γ_i is a forgetting factor adjusting the influence of recent events in comparison to earlier events with $0 \leq \gamma \leq 1$. Hence, model 1, including an individual variance parameter (see *Model Parametrization* below), contains 5 parameters per participant.

We fit an alternative model containing a term for the gamble U to verify that the gamble uncertainty influences affective experience (model 2), which improved the model fit (see Table S1).

$$AE_{t,i} = w_{0,i} + w_{EV,i} \sum_{j=1}^{t} {\gamma_i}^{t-j} * EV_{j,i} + w_{U,i} \sum_{j=1}^{t} {\gamma_i}^{t-j} * U_{j,i} + w_{PE,i} \sum_{j=1}^{t} {\gamma_i}^{t-j} * PE_{j,i} \quad \text{(S.2)}$$

To verify that affective experience depends on expectations and prediction errors rather than actual outcomes, we next fit a model containing a term for the outcome received on each trial instead of expectations and prediction errors (model 3). Model 3 indicated that expectations and prediction errors explain more variance in affective experience than actual outcomes (Table S1). Additionally, we fit two additional models (variations of model 2) that included different parametrizations of PE, which both improved the model fit compared to model 2 (Table S1). Model 4 included separate terms for positive and negative PE to capture the differential influence of positive and negative surprise on valence and arousal (see Equation S3). Model 5 included signed and unsigned PE terms, which captures the impact of the magnitude of PE independent of its direction (Equation S4). This model was included to

investigate whether affective experience (especially the arousal feature of affect) is more sensitive to the size of PE rather than its direction.

Model 4

$$AE_{t,i} = w_{0,i} + w_{EV,i}\sum_{j=1}^{t}{\gamma_i}^{t-j} * EV_{j,i} + w_{U,i}\sum_{j=1}^{t}{\gamma_i}^{t-j} * U_{j,i} + w_{posPE,i}\sum_{j=1}^{t}{\gamma_i}^{t-j} * posPE_{j,i} + w_{negPE,i}\sum_{j=1}^{t}{\gamma_i}^{t-j} * negPE_{j,i} \quad (S.3)$$

Model 5

$$AE_{t,i} = w_{0,i} + w_{EV,i}\sum_{j=1}^{t}{\gamma_i}^{t-j} * EV_{j,i} + w_{U,i}\sum_{j=1}^{t}{\gamma_i}^{t-j} * U_{j,i} + w_{PE,i}\sum_{j=1}^{t}{\gamma_i}^{t-j} * PE_{j,i} + w_{|PE|,i}\sum_{j=1}^{t}{\gamma_i}^{t-j} * |PE_{j,i}| \quad (S.4)$$

The model fit of these five different models indicated that model 5 provided the best fit for both valence and arousal ratings (Table S2), which suggests that momentary affective experience reflects temporal integration of expectations, uncertainty, and the direction and magnitude of prediction errors.

Table S1. The model fits for different affective experience models.

**Model no**	**Parameters per individual**	**Model WAIC**		**WAIC – WAIC$_{model1}$**	
		Valence	Arousal	Valence	Arousal
1	5	10889	12958	0	0
2	6	10821	12780	-68	-178
3	5	10924	13460	35	502
4	7	10764	12458	-125	-500
5	7	10736	12406	-153	-552

Model parametrization and fit

Individual-level parameters were drawn from group-level normal distributions. Additionally, we used a non-centered parametrization to speed up estimation (Ahn et al., 2017; Stan Development Team, 2018). All the weight parameters, including the constant term, were parameterized as follows:

$$\begin{gathered}\mu_w \sim Normal(0,1)\\ \sigma_w \sim Halfnormal(0,0.2)\\ \acute{w} \sim Normal(0,1)\\ w = \mu_w + \sigma_w.\acute{w}\end{gathered} \tag{S5}$$

Here, $\acute{w}$ represents individual-level variations from the group mean (μ_w), and w in Equation S5 is mathematically equivalent to $w \sim Normal(\mu_w, \sigma_w)$.

The forgetting factor (γ) was parametrized similarly with only difference being the probit transformation (the cumulative distribution function of a unit normal distribution) of the individual level parameters to ensure $0 \leq \gamma \leq 1$.

$$\begin{gathered}\mu_\gamma \sim Normal(0,1)\\ \sigma_\gamma \sim Halfnormal(0,0.2)\\ \acute{\gamma} \sim Normal(0,1)\\ \gamma = Probit^{-1}(\mu_\gamma + \sigma_\gamma.\acute{\gamma})\end{gathered} \tag{S6}$$

The observed valence and arousal ratings in the experiment were modeled using a normal distribution, $Normal(\mu_{i,t},\ \sigma_i)$. $\mu_{i,t}$ is the individual specific location changing in every trial and calculated according to the model (Equation S4). σ_i is the individual standard deviation sampled as follows (here the exponential transformation ensures a positive value for the individual level variance).

$$\begin{gathered}\mu_\sigma \sim Normal(0,1)\\ \sigma_\sigma \sim Halfnormal(0,0.2)\\ \acute{\sigma} \sim Normal(0,1)\\ \sigma = exp(\mu_\sigma + \sigma_\sigma.\acute{\sigma})\end{gathered} \tag{S7}$$

Choice model

Parameter recovery

We modeled the choice behavior based on subjective utility computations with two parameters: loss aversion (λ) and risk sensitivity (ρ). The softmax choice rule with an inverse temperature parameter (i.e. choice consistency; c) was used to compute the probability of accepting the gamble (Equations 5-7 in the main manuscript). We used hierarchical Bayesian analysis to estimate group and individual level parameters (see below for *Model Parametrization and Fit*). Since we used a novel risky choice task, we carried out a parameter recovery analysis to ensure that the study and the modeling procedure can provide reliable results under ideal conditions.

We simulated 100 datasets (each with 101 subjects) randomly selecting group level parameters according to the following:

$$\begin{gathered} \mu_\lambda \sim Uniform(0.5, 3) \\ \mu_\rho \sim Uniform(0.4, 2) \\ \mu_c \sim Uniform(0, 5) \\ \sigma_\lambda \sim Exponential(0.12) \\ \sigma_\rho \sim Exponential(0.08) \\ \sigma_c \sim Exponential(0.25) \end{gathered} \tag{S8}$$

This selection ensured a wide range of group level parameters, which were used to select individual level parameters (N=10100 in total) and generate individual data. Then, the choice model was fit to each dataset using HBA to estimate both group and individual level parameters. Figure S3 shows the correlations between simulated and estimated parameters. The results show that group level means for ρ (Pearson-R = .998) and λ (Pearson-R = .999) were recovered reliably. The choice consistency parameter was recovered reliably within 0 to 2 range. However, around 30% of simulations, in which c was higher than 2, were overestimated (Pearson-R = .67). The individual level parameters for ρ (Pearson-r = .988) and λ (Pearson-r = .996) were also recovered reliably. We also found that 95% HDIs of the posterior distribution of parameters contained the original parameter values generating the dataset in 93% of the simulations. In addition, Table S2 summarizes correlations between estimated parameters, which indicates that ρ and λ was recovered independently. Taken together, these simulations indicate that loss aversion and risk sensitivity parameters could be recovered independently over a reasonable range under ideal conditions, while the choice consistency could be recovered reliably within the range from 0 to 2.

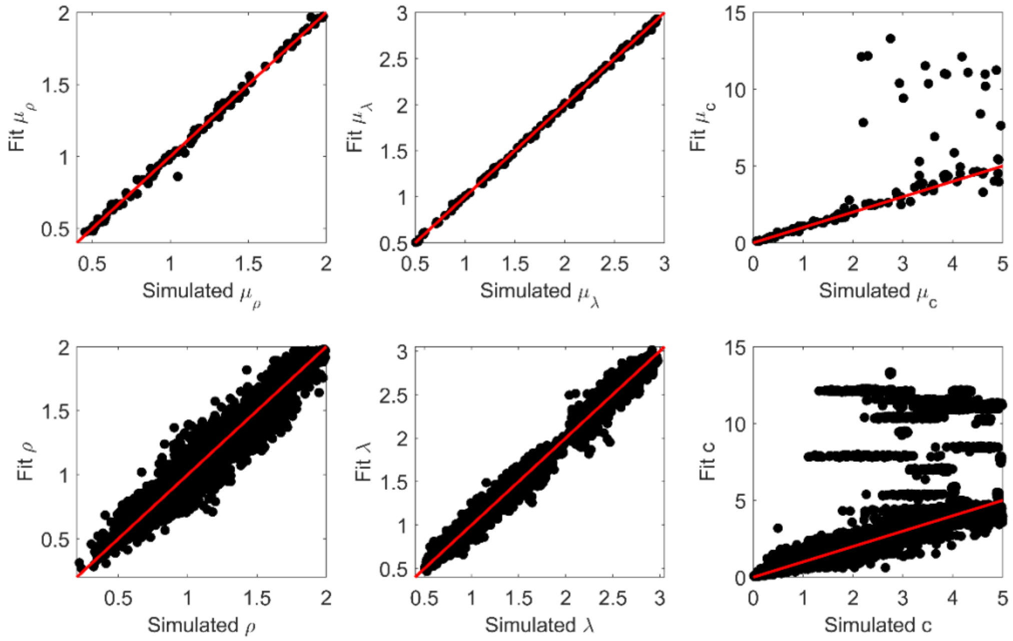

Figure S3. Parameter recovery for the choice model. Top row shows the correlation between simulated and estimated group level means for ρ, λ, and c (100 simulated groups). Bottom row shows the correlation between simulated and estimated ρ, λ, and c at the individual level (10100 individuals). ρ = risk sensitivity; λ = loss aversion; c = choice consistency.

Table S2. Pearson correlations between estimated group level parameters.

	$\boldsymbol{\mu_\rho}$	$\boldsymbol{\mu_\lambda}$
$\boldsymbol{\mu_\lambda}$	.06 (ns.)	
$\boldsymbol{\mu_c}$	.42 (p<.05)	-.05 (ns.)

Next, we investigated the parameter combinations to identify the cases in which $\boldsymbol{\mu_c}$ is overestimated. We found that the combination of $\boldsymbol{\mu_\rho} > 1$ (which implies increasing marginal utility) and $\boldsymbol{\mu_c} > 2$ resulted in the overestimation of $\boldsymbol{\mu_c}$ (Figure S4). To verify this finding, we simulated 1000 data points with several combinations of $\boldsymbol{\mu_\rho}$ and $\boldsymbol{\mu_c}$ (with $\boldsymbol{\mu_\lambda}=1$). Figure S5 shows the expected value of the gambles against the probability of accepting for various data generating parameters. The simulations verify that when the risk sensitivity parameter is large different choice consistency levels do not differentiate. Taken together, these findings suggest

that the current task design and analysis is limited when the data generating process is defined by increasing marginal utility and high choice consistency.

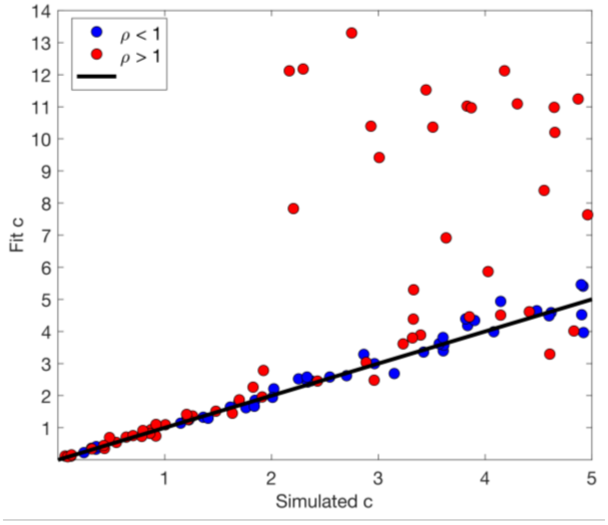

Figure S4. The correlation between simulated and estimated group level mean for the choice consistency parameter plotted according to the data generating risk sensitivity parameter. ρ = risk sensitivity; c = choice consistency.

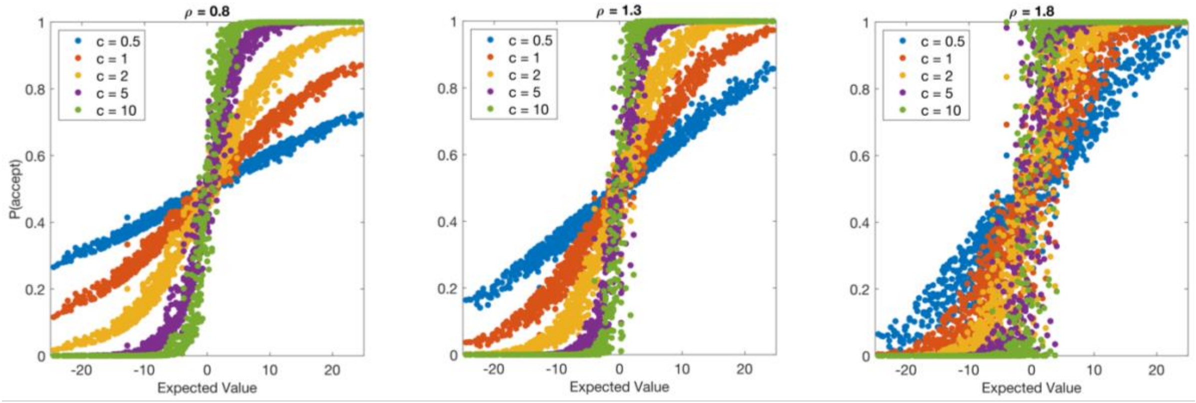

Figure S5. Simulated data sets with several combinations of risk sensitivity (ρ) and choice consistency (c) parameters showing Expected Value of the gamble with respect to the probability of accepting the given gamble. 1000 datasets were simulated with each ρ and c combination. Loss aversion is set to 1.

Model parametrization and fit

We used a non-centered parametrization and individual-level parameters were drawn from group-level normal distributions. Loss aversion was parameterized as follows:

$$\begin{gathered}\mu_\lambda \sim Normal(0,1)\\ \sigma_\lambda \sim Halfnormal(0,0.2)\\ \acute{\lambda} \sim Normal(0,1)\\ \lambda = Probit^{-1}(\mu_\lambda + \sigma_\lambda.\acute{\lambda}) * 5\end{gathered} \tag{S4}$$

We used $\lambda = Probit^{-1}(\mu_\lambda + \sigma_\lambda.\acute{\lambda}) * 5$ to ensure that $0 \leq \lambda \leq 5$. The other parameters, ρ and c, were parametrized similarly with the only differences being $\rho = Probit^{-1}(\mu_\rho + \sigma_\rho.\acute{\rho}) * 2$ and $c = Probit^{-1}(\mu_c + \sigma_c.\acute{c}) * 20$ to ensure $0 \leq \rho \leq 2$ *and* $0 \leq c \leq 20$.

Influence of affective experience. We introduced regression coefficients to model the degree to which trial-by-trial variations in affective experience influences decision parameters (see Eq8 in the main text). The non-centered parametrization of the regressions was done in the following manner with weekly informative priors on the regression coefficients.

$$\begin{gathered}\mu_\lambda \sim Normal(0,1)\\ \sigma_\lambda \sim Halfnormal(0,0.2)\\ \acute{\lambda}_i \sim Normal(0,1)\\ \lambda_i = \mu_\lambda + \sigma_\lambda.\acute{\lambda}_i\end{gathered} \tag{S5}$$

$$\begin{gathered}\mu_{\beta A} \sim Normal(0,1)\\ \sigma_{\beta A} \sim Halfnormal(0,0.2)\\ \acute{\beta}_{A,i} \sim Normal(0,1)\\ \beta_{A,i} = \mu_{\beta A} + \sigma_{\beta A}.\acute{\beta}_{A,i}\end{gathered}$$

$$\begin{gathered}\mu_{\beta V} \sim Normal(0,1)\\ \sigma_{\beta V} \sim Halfnormal(0,0.2)\\ \acute{\beta}_{V,i} \sim Normal(0,1)\\ \beta_{V,i} = \mu_{\beta V} + \sigma_{\beta V}.\acute{\beta}_{V,i}\end{gathered}$$

$$\lambda_{i,t} = 5 * Probit^{-1}\big(\lambda_i + \beta_{A,i}.Arousal_i(t) + \beta_{V,i}.Valence_i(t)\big)$$

Model fitting. We first estimated the parameters of the choice model without the affective influences (Figure S6). The posterior distribution of the group level parameters suggested that participants on average demonstrated risk averse behavior (95% HDI on ρ = [0.5, 0.61]). Additionally, there was evidence for no loss aversion as the 95% HDI on λ

included 1 (95% HDI on λ = [0.96, 1.09]). Finally, participants were not random in their choices (95% HDI on c = [1.44, 2.07]).

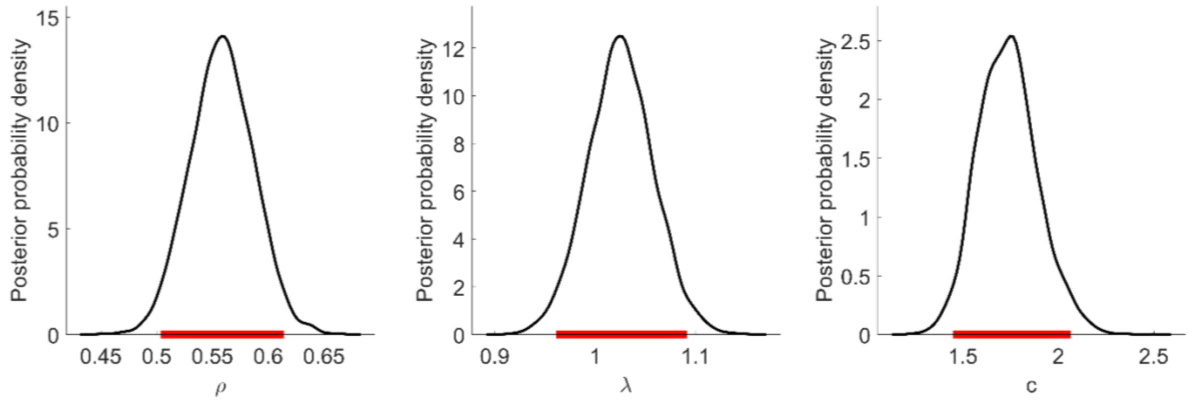

Figure S6. The posterior distribution on the parameter estimates of the base choice model.

Next, we fit a full model, in which experienced valence and arousal influence trial-by-trial variations in all decision parameters (Figure S7). This model resulted in similar group level means to the base model. Additionally, the results showed that valence did not reliably influenced any of the parameters, while arousal was negatively associated with ρ (95% HDI on $\beta_A \propto \rho$ = [-0.15, -0.05]), and positively associated with both λ (95% HDI on $\beta_A \propto \lambda$ = [0.01, 0.07]) and c (95% HDI on $\beta_A \propto c$ = [0.1, 0.81]). This means that increased trial-to-trial arousal leads to slightly higher loss aversion, higher risk aversion, and higher choice-consistency (Figure S5). According to WAIC, full model performed better than the base model without trial-by-trial variations, $WAIC_{Full}$=2698; $WAIC_{Base}$=2714. Next, we removed valence modulators from the full model to see whether the model fit would improve. The results showed that this adjusted model performed slightly better than the full model ($WAIC_{Adjusted}$=2694), and the posterior distributions of the regressors indicated that experienced arousal before the choice influenced loss-aversion, risk-aversion, and choice-consistency (Figure 4 in the main text).

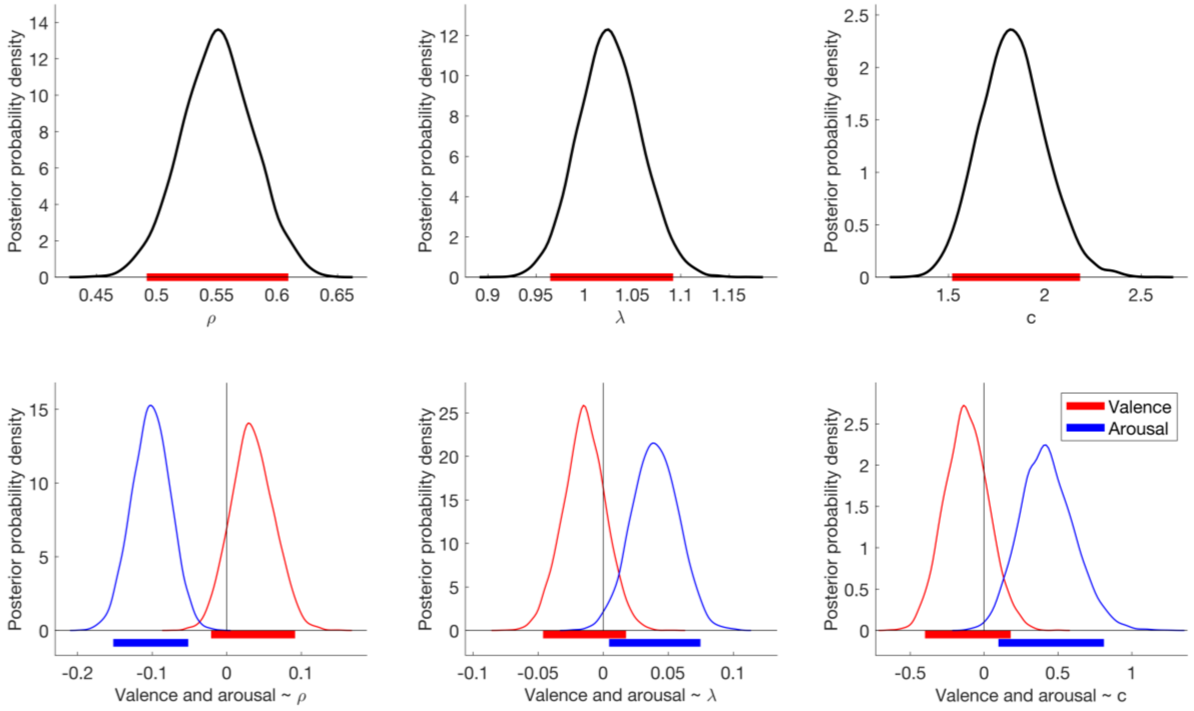

Figure S7. The posterior distribution on the parameter estimates of the full choice model including trial-to-trial influences of valence and arousal on decision parameters. Upper panel shows the posterior distributions and the 95% HDIs for group level parameters. The lower panel shows the posterior distribution of the regression coefficients.

In addition, we fit two models in which the effect of experienced arousal was tested while controlling for the previous outcome (control model 1) and previous PE (control model 2). In these models, both experienced arousal and either the previous outcome or the previous PE are allowed to modulate the decision parameters. The results showed that the modulatory effect of arousal on all decision parameters persisted when controlling for previous gamble outcome and PE. While the influence of the gamble outcome and PE from the previous trial was not different from zero.

Table S3. The 95% HDIs for the regression coefficients and model information criteria for the control models.

Control model 1		Control Model 2	
$\beta_A \propto \rho$	[-0.108, -0.028]	$\beta_A \propto \rho$	[-0.108, -0.028]
$\beta_A \propto \lambda$	[0.004, 0.064]	$\beta_A \propto \lambda$	[0.007, 0.065]
$\beta_A \propto c$	[0.004, 0.536]	$\beta_A \propto c$	[0.011, 0.524]
$\beta_{outcome} \propto \rho$	[-0.032, 0.039]	$\beta_{PE} \propto \rho$	[-0.023, 0.053]
$\beta_{outcome} \propto \lambda$	[-0.017, 0.023]	$\beta_{PE} \propto \lambda$	[-0.035, 0.008]
$\beta_{outcome} \propto c$	[-0.195, 0.192]	$\beta_{PE} \propto c$	[-0.241, 0.165]
WAIC	2705	WAIC	2702

References

Ahn, W. Y., Haines, N., & Zhang, L. (2017). Revealing neurocomputational mechanisms of reinforcement learning and decision-making with the hBayesDM package. *Computational Psychiatry* **1**, 24-57. DOI: https://doi.org/10.1162/CPSY_a_00002

Stan Development Team. (2018). RStan: The R interface to Stan. http://mc-stan.org.

Table S4. Complete choice set for an individual in the study.

Trial no	Outcome 1	Outcome 2	Outcome 3	Outcome 4	Gamble EV
1	'LOSE 5kr @ 35%'	'WIN 45kr @ 25%'	'WIN 10kr @ 5%'	'LOSE 35kr @ 35%'	-2.25 kr
2	'LOSE 25kr @ 25%'	'LOSE 30kr @ 45%'	'WIN 5kr @ 15%'	'LOSE 10kr @ 15%'	-20.5 kr
3	'LOSE 10kr @ 35%'	'WIN 10kr @ 25%'	'LOSE 25kr @ 15%'	'WIN 15kr @ 25%'	-1 kr
4	'WIN 25kr @ 45%'	'WIN 10kr @ 25%'	'WIN 5kr @ 5%'	'LOSE 15kr @ 25%'	10.25 kr
5	'LOSE 5kr @ 15%'	'LOSE 10kr @ 15%'	'WIN 5kr @ 35%'	'WIN 15kr @ 35%'	4.75 kr
6	'WIN 15kr @ 25%'	'WIN 25kr @ 35%'	'WIN 5kr @ 15%'	'LOSE 25kr @ 25%'	7 kr
7	'WIN 5kr @ 25%'	'LOSE 5kr @ 35%'	'LOSE 35kr @ 15%'	'WIN 20kr @ 25%'	-0.75 kr
8	'LOSE 25kr @ 25%'	'LOSE 15kr @ 35%'	'LOSE 5kr @ 15%'	'WIN 20kr @ 25%'	-7.25 kr
9	'LOSE 10kr @ 5%'	'WIN 15kr @ 25%'	'WIN 10kr @ 45%'	'WIN 5kr @ 25%'	9 kr
10	'LOSE 15kr @ 25%'	'WIN 15kr @ 25%'	'WIN 5kr @ 5%'	'LOSE 10kr @ 45%'	-4.25 kr
11	'LOSE 35kr @ 15%'	'WIN 30kr @ 5%'	'LOSE 25kr @ 35%'	'WIN 15kr @ 45%'	-5.75 kr
12	'WIN 10kr @ 15%'	'WIN 30kr @ 45%'	'LOSE 20kr @ 5%'	'LOSE 15kr @ 35%'	8.75 kr
13	'WIN 30kr @ 35%'	'LOSE 15kr @ 45%'	'WIN 15kr @ 15%'	'WIN 20kr @ 5%'	7 kr
14	'WIN 5kr @ 45%'	'LOSE 5kr @ 35%'	'LOSE 15kr @ 15%'	'WIN 25kr @ 5%'	-0.5 kr
15	'LOSE 45kr @ 15%'	'LOSE 10kr @ 15%'	'WIN 10kr @ 45%'	'WIN 40kr @ 25%'	6.25 kr
16	'LOSE 5kr @ 25%'	'WIN 5kr @ 5%'	'LOSE 10kr @ 45%'	'LOSE 25kr @ 25%'	-11.75 kr
17	'LOSE 20kr @ 45%'	'LOSE 10kr @ 25%'	'LOSE 15kr @ 25%'	'LOSE 5kr @ 5%'	-15.5 kr
18	'LOSE 10kr @ 25%'	'WIN 40kr @ 45%'	'WIN 5kr @ 15%'	'WIN 10kr @ 15%'	17.75 kr
19	'LOSE 35kr @ 15%'	'LOSE 25kr @ 25%'	'WIN 15kr @ 25%'	'LOSE 10kr @ 35%'	-11.25 kr
20	'LOSE 25kr @ 15%'	'LOSE 10kr @ 35%'	'LOSE 5kr @ 35%'	'WIN 35kr @ 15%'	-3.75 kr
21	'LOSE 15kr @ 45%'	'LOSE 5kr @ 15%'	'WIN 30kr @ 5%'	'LOSE 25kr @ 35%'	-14.75 kr
22	'WIN 15kr @ 25%'	'LOSE 10kr @ 35%'	'WIN 10kr @ 35%'	'WIN 30kr @ 5%'	5.25 kr
23	'WIN 25kr @ 35%'	'LOSE 15kr @ 15%'	'WIN 5kr @ 5%'	'WIN 10kr @ 45%'	11.25 kr
24	'LOSE 10kr @ 25%'	'WIN 10kr @ 15%'	'LOSE 15kr @ 25%'	'WIN 20kr @ 35%'	2.25 kr
25	'LOSE 5kr @ 45%'	'LOSE 30kr @ 25%'	'LOSE 20kr @ 5%'	'WIN 10kr @ 25%'	-8.25 kr
26	'LOSE 35kr @ 5%'	'WIN 5kr @ 35%'	'WIN 45kr @ 35%'	'LOSE 10kr @ 25%'	13.25 kr
27	'WIN 40kr @ 35%'	'LOSE 30kr @ 25%'	'LOSE 25kr @ 25%'	'WIN 10kr @ 15%'	1.75 kr

28	'WIN 10kr @ 15%'	'WIN 25kr @ 15%'	'LOSE 30kr @ 65%'	'WIN 20kr @ 5%'	-13.25 kr
29	'WIN 40kr @ 25%'	'LOSE 35kr @ 35%'	'LOSE 5kr @ 5%'	'WIN 25kr @ 35%'	6.25 kr
30	'WIN 5kr @ 25%'	'LOSE 5kr @ 25%'	'LOSE 10kr @ 35%'	'LOSE 20kr @ 15%'	-6.5 kr
31	'LOSE 15kr @ 15%'	'WIN 35kr @ 15%'	'WIN 10kr @ 35%'	'LOSE 25kr @ 35%'	-2.25 kr
32	'WIN 30kr @ 35%'	'WIN 5kr @ 15%'	'WIN 15kr @ 25%'	'LOSE 10kr @ 25%'	12.5 kr
33	'WIN 10kr @ 15%'	'WIN 5kr @ 25%'	'LOSE 30kr @ 35%'	'WIN 25kr @ 25%'	-1.5 kr
34	'LOSE 30kr @ 15%'	'LOSE 5kr @ 45%'	'WIN 30kr @ 35%'	'WIN 5kr @ 5%'	4 kr
35	'WIN 30kr @ 25%'	'WIN 5kr @ 25%'	'LOSE 10kr @ 15%'	'LOSE 20kr @ 35%'	0.25 kr
36	'LOSE 10kr @ 25%'	'LOSE 5kr @ 35%'	'LOSE 35kr @ 15%'	'LOSE 40kr @ 25%'	-19.5 kr
37	'LOSE 20kr @ 15%'	'WIN 15kr @ 15%'	'LOSE 5kr @ 35%'	'WIN 20kr @ 35%'	4.5 kr
38	'WIN 10kr @ 25%'	'LOSE 20kr @ 15%'	'LOSE 15kr @ 25%'	'WIN 15kr @ 35%'	1 kr
39	'WIN 10kr @ 35%'	'WIN 25kr @ 5%'	'LOSE 15kr @ 45%'	'LOSE 10kr @ 15%'	-3.5 kr
40	'LOSE 5kr @ 25%'	'LOSE 30kr @ 25%'	'WIN 10kr @ 35%'	'LOSE 20kr @ 15%'	-8.25 kr
41	'LOSE 10kr @ 15%'	'WIN 25kr @ 25%'	'LOSE 30kr @ 25%'	'WIN 5kr @ 35%'	-1 kr
42	'WIN 5kr @ 35%'	'LOSE 5kr @ 5%'	'LOSE 10kr @ 25%'	'LOSE 20kr @ 35%'	-8 kr
43	'LOSE 35kr @ 35%'	'LOSE 10kr @ 25%'	'WIN 5kr @ 15%'	'LOSE 30kr @ 25%'	-21.5 kr
44	'LOSE 25kr @ 15%'	'WIN 30kr @ 45%'	'LOSE 5kr @ 25%'	'WIN 5kr @ 15%'	9.25 kr
45	'WIN 5kr @ 25%'	'LOSE 10kr @ 45%'	'LOSE 30kr @ 5%'	'WIN 10kr @ 25%'	-2.25 kr
46	'LOSE 5kr @ 15%'	'WIN 25kr @ 45%'	'LOSE 25kr @ 35%'	'LOSE 10kr @ 5%'	1.25 kr
47	'LOSE 5kr @ 25%'	'WIN 5kr @ 5%'	'WIN 25kr @ 45%'	'WIN 35kr @ 25%'	19 kr
48	'WIN 25kr @ 35%'	'LOSE 5kr @ 5%'	'LOSE 15kr @ 45%'	'WIN 10kr @ 15%'	3.25 kr
49	'LOSE 5kr @ 15%'	'WIN 35kr @ 45%'	'WIN 5kr @ 15%'	'WIN 15kr @ 25%'	19.5 kr
50	'WIN 45kr @ 25%'	'LOSE 40kr @ 25%'	'LOSE 5kr @ 25%'	'WIN 30kr @ 25%'	7.5 kr
